# Identifying N^6^-methyladenosine sites using multi-interval nucleotide pair position specificity and support vector machine

**DOI:** 10.1038/srep46757

**Published:** 2017-04-25

**Authors:** Pengwei Xing, Ran Su, Fei Guo, Leyi Wei

**Affiliations:** 1 School of Computer Science and Technology, Tianjin University, Tianjin, China; 2 School of Software, Tianjin University, Tianjin, China

## Abstract

N6-methyladenosine (m^6^A) refers to methylation of the adenosine nucleotide acid at the nitrogen-6 position. It plays an important role in a series of biological processes, such as splicing events, mRNA exporting, nascent mRNA synthesis, nuclear translocation and translation process. Numerous experiments have been done to successfully characterize m^6^A sites within sequences since high-resolution mapping of m^6^A sites was established. However, as the explosive growth of genomic sequences, using experimental methods to identify m^6^A sites are time-consuming and expensive. Thus, it is highly desirable to develop fast and accurate computational identification methods. In this study, we propose a sequence-based predictor called RAM-NPPS for identifying m^6^A sites within RNA sequences, in which we present a novel feature representation algorithm based on multi-interval nucleotide pair position specificity, and use support vector machine classifier to construct the prediction model. Comparison results show that our proposed method outperforms the state-of-the-art predictors on three benchmark datasets across the three species, indicating the effectiveness and robustness of our method. Moreover, an online webserver implementing the proposed predictor has been established at http://server.malab.cn/RAM-NPPS/. It is anticipated to be a useful prediction tool to assist biologists to reveal the mechanisms of m^6^A site functions.

N^6^-methyladenosine (m^6^A) is firstly found in polyadenylated RNA from mammalian cells in the 1970s^1^,^2^,^3^,^4^. Since then, m^6^A is observed in many species, such as *bacteria, Homo sapiens, Arabidopsis thaliana,* and *Saccharomyces cerevisiae,* etc. It is currently the most hot topic among ~150 RNA-modification types^5^. m^6^A involves many molecular processes, including brain development abnormalities and other diseases^6^, protein translation and localization^7^, and even contributed to obesity^8^. Recent studies have suggested that the regions in 5′ untranslated regions (UTRs), around stop codons and in 3′ UTRs neighbor stop codons has a number of m^6^A residues^9^,^10^, indicating that m^6^A exists high specificity in these regions. Thus, accurate identification of m^6^A sites is the first step to provide in-depth understanding of their biological functions.

In the last few decades, many computational methods have developed for the identification of m^6^A sites. Researchers use the motif discovery algorithm and find that m^6^A peaks has a consensus motif with form of DRACH (where D = A, G or U; R = A or G; H = A, C or U)^11^,^12^,^13^,^14^,^15^. These results show m^6^A writers which refer to adenosine methyltransferases including METTL3, METTL14, WTAP, and KIAA1429, and m^6^A erasers which refers to that demethylases including FTO and ALKBH5 may constitute a limited repertoire with predominant and a few less abundant elements^16^. At the same time, there are a mass of consensus motifs that are not methylated. To identify methylated m^6^A sites, it is imperative to build a high-resolution data for predicting m^6^A sites. Schwartz *et al*. constructed a single-nucleotide resolution genomic map of m^6^A sites in the *Saccharomyces cerevisiae* species^13^. Using this high resolution data, Chen *et al*. proposed a predictor called “iRNA-Methyl”, which formulates RNA sequences by using “pseudo dinucleotide composition” together with three RNA physiochemical properties to make predictions^17^,^18^. Jaffrey *et al*. built a single-nucleotide resolution map of m^6^A sites across *Homo sapiens*^14^. Zhou and his co-workers developed a mammalian m^6^A site predictor called SRAMP, which proposed three feature encoding algorithms, such as positional binary encoding of nucleotide sequence, the K-nearest neighbor (KNN) encoding, and the nucleotide pair spectrum encoding^19^. More recently, Chen *et al*. proposed a support vector machine-based method to predict m^6^A sites in *Arabidopsis thaliana*^20^. In some studies, well-established ensemble classifiers are proved to outperform single classifiers^21^,^22^,^23^. Based on this, Chen *et al*. thus proposed a m^6^A predictor by constructing an ensemble classifier based on support vector machine to successfully improve the predictive performance^24^.

Although many computational efforts have been done in the prediction of m^6^A sites, existing methods are still far from being accurate. The major difficulty is that feature representation algorithms are not informative enough to capture insight differences between true m^6^A sites and non-m^6^A sites^25^, thus resulting in the low discriminatory ability of feature representations. In this study, we propose a novel feature representation algorithm, in which we sufficiently capture both the global and local information based on multi-interval nucleotide pair position specificity, and successfully convert RNA sequences into high-quality feature representations. Using the proposed feature representations and support vector machine (SVM), we propose a sequence-based predictor called RAM-NPPS for identifying m^6^A sites, where “R” stands for RNA, “A” stands for N^6^-adenosine, “M” stands for methylation, and “NPPS” stands for nucleotide pair position specificity. Comprehensive comparison results on three benchmark datasets across three species show that our proposed RAM-NPPS performs remarkably better than the state-of-the-art predictors. For academic convenience, we establish an online webserver implementing the proposed predictor at http://server.malab.cn/RAM-NPPS/.

## Materials and Methods

### Datasets

As indicated in many previous studies, datasets are fundamentally important to build a robust and accurate prediction model^26^,^27^. In this study, we employed three benchmark datasets across three species to comprehensively evaluate the performance of the proposed predictor. The details of the three datasets are described as follows.

#### Saccharomyces cerevisiae dataset

This dataset is originally proposed by Chen *et al*.^28^. The dataset contains 1,307 positive sequences with m^6^A sites and 1,307 negative sequences with non-m^6^A sites. It is worth noting that the negative samples are randomly collected from 33,280 sequences with non-m^6^A sites. All sequences in the dataset are 51-nt long (25-nt on each side of the m^6^A/non-m^6^A sites) with the sequence similarity less than 85%.

#### Homo sapiens dataset

This dataset, downloaded from Zhou’s work^19^, recompiles the recently published single-nucleotide resolution maps of mammalian m^6^A sites^14^. The dataset contains 8,366 positive samples and the equal number of negative samples. The negative samples are selected from 65,345 negative samples randomly. All sequences in this dataset are 51-nt long as well.

#### Arabidopsis thaliana dataset

This benchmark dataset is downloaded from Chen’s study^20^. The dataset contains 394 positive samples and the same number of negative samples. The sequences in this dataset share less than 60% sequence similarity.

For academic convenience, we provide all the three datasets mentioned above in our webserver. They are freely available to be downloaded from the following website: http://server.malab.cn/RAM-NPPS/data.jsp.

### Framework of the proposed predictor

**Figure 1:**
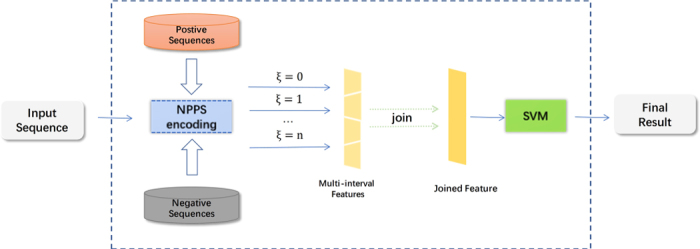
Overall framework of the proposed predictor.

Figure 1 illustrates the overall framework of the RAM-NPPS method for m^6^A site prediction. The prediction process of the proposed RAM-NPPS predictor is described as follows. Firstly, input sequences are encoded by the proposed NPPS (nucleotide pair position specificity) feature representation algorithm to obtain the meaningful feature vectors. Then, the resulting feature vectors with different parameter (ξ) values are joined together into one. Finally, the joined ones are fed into the SVM classifier to make predictions.

### Feature encoding algorithm

For convenience of discussion, the dataset can be denoted as,


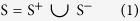


where S is the entire dataset; S^+^ is the set of all positive samples, i.e., all RNA sequences containing m^6^A sites; S^−^ is the set of all negative samples, i.e., all RNA sequences containing nonk-m^6^A sites.

For a given RNA sequence, it can be encoded with the following formula:


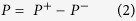


where *P*^+^ is formulated as:


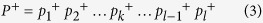


where *p*_*k*_ represents the *k*-th nucleotide, *l* is the length of the sequence.

To calculate *p*_*k*_^+^, let us define two matrices T_*s*_^+^ and T_*d*_^+^ :


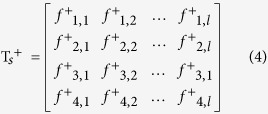


where rows represent {*A, C, G, U*}, respectively; column represents the length of the sequence. The element *f*^+^_1,1_ represents the single nucleotide occurrence probability of the ‘A’ nucleotide in all positive sequences (samples) at the 1^st^ position of the sequence for example.


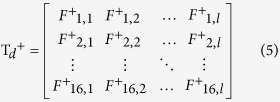


where rows represent {A C G U} × {A C G U}, respectively; column represents the length of the RNA sequence; the element *F*^+^_1,2_ represents the occurrence probability of the nucleotide pair ‘AC’ in all positive samples at the position of 2-nd and (2 + ξ)-th nucleotide of the RNA sequence, where ξ is the interval of the two nucleotides in a pair. It is worth noting that ξ = 0 denotes the continuous dinucleotide.

Assuming that the dinucleotide between the *k*-th nucleotide and (k + ξ)-th nucleotide is ‘CG’, *p*_*k*_^+^ can be computed the following formula by using the conditional probability formula 
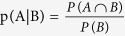
,


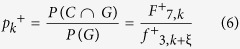


where 7 is the index of ‘CG’ in the {A C G U} × {A C G U}, and 3 is the index of ‘G’ in the {A C G U}.

Accordingly, we obtained *P*^+^ from S^+^. Similarly, we obtained *P*^−^ from S^−^. Finally, the RNA sequence is successfully converted into the feature vector *P* by formula (2).

**Figure 2:**
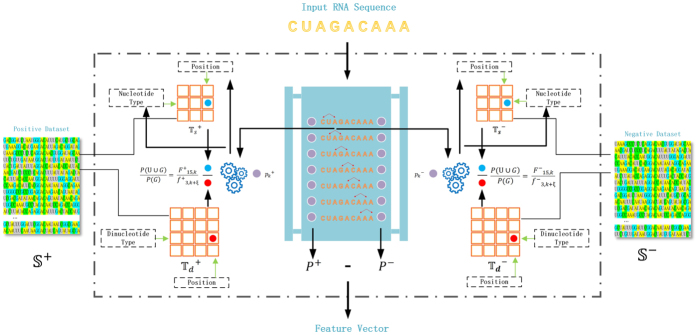
Schematic workflow of the proposed feature encoding scheme.

Figure 2 shows the NPPS feature representation process. Firstly, we compute nucleotide position specificity information by counting the occurrence frequency of different nucleotide types at different positions for the positive dataset S^+^and the negative sequence set S^−^, respectively. Then, the information is stored in matrices T_*s*_^+^, T_*d*_^+^, T_*s*_^−^, and T_*d*_^−^. T_*s*_^+^ stores single nucleotide position specificity information of the positive sequences and T_*d*_^+^ stores nucleotide pair position specificity information of the positive sequences, T_*s*_^−^and T_*d*_^−^ are for negative sequences. When it comes to an input sequence, we can get *P*^+^ and *P*^−^ of the input sequence according to the four matrices above. Finally, we successfully encode the input sequence into a feature vector by the subtraction of *P*^+^ and *P*^−^.

In the above process, we can obtain the local sequential information by setting the parameter ξ and getting multi-interval nucleotide pair position information within the sequence. This makes our features reflect relevance of different interval nucleotides. Moreover, by counting frequency of nucleotide position in entire positive dataset and negative dataset, we can get the global information between positive and negative samples.

### Support Vector Machine (SVM)

Support Vector Machine (SVM) is a supervised machine learning method based on statistical theory. Due to its high efficacy for classification task, SVM has been widely applied into bioinformatics^29^,^30^,^31^,^32^,^33^,^34^,^35^. In brief, the algorithm of SVM is to transform sample data with different classes into a high-dimension feature space, and then learn an optimal decision boundary or hyper plane for the data from different classes using kernel functions.

In this study, the LibSVM package (http://www.csie.ntu.edu.tw/~cjlin/libsvm/) is employed, which is an implementation of SVM. Radial Basis Function (RBF) is set as the kernel function of SVM. Moreover, there are two parameters (penalty constant C and width) in the SVM algorithm. To build a SVM model with high-level performance, the two parameters are optimized by using the grid search approach based on F-score, which considers both precision and recall of the test to evaluate the two parameters.

### Evaluation Metrics

In binary predictors, four metrics are usually used to measure the predictive performance, including sensitivity (Sn), specificity (Sp), Accuracy (Acc), and the Mathew’s correlation coefficient (*MCC*), respectively. In this study, the four metrics are employed to evaluate the performance of m^6^A predictors (binary predictor) as well. They are formulated as:


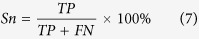



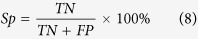



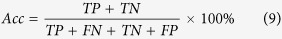



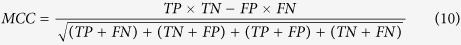


where TP, TN, FP and FN is the number of true positive, true negative, false positive, and false negative, respectively. In current study, TP represents the total number of the RNA fragment sequences centered with true m^6^A sites that are predicted as m^6^A sequences correctly; TN represents the total number of the RNA fragment sequences centered with non-m^6^A sites that are predicted as non-m^6^A sequences correctly; FP represents the number of those non-m^6^A sequences that are recognized as m^6^A sequences while FN represents the number of those m^6^A sequences that are recognized as non-m^6^A sequences.

### Evaluation Methods

In this study, we employ the *k*-fold cross-validation method to evaluate the performance of m^6^A predictors. In *k*-fold cross-validation, a dataset is randomly partitioned into *k* subsets. Of the *k* subsets, a single subset is retained as the validation data for testing the model, and the remaining *k* − 1 subsets are used as training data. The cross-validation process is then repeated *k* times (the folds), with each of the *k* subsets used exactly once as the validation data. The *k* results from the folds then can be averaged (or otherwise combined) to produce a final performance estimation. 10-fold cross-validation is commonly used.

## Results and Discussion

### Impact of the parameter ξ

In the proposed NPPS feature algorithm, there is a parameter ξ that describes the interval of the nucleotide pairs. Varying the ξ value generates various features, thereby impacting the predictive performance. To investigate the effect of the parameter ξ, we discuss the performance of models based on features over different values of ξ. Theoretically speaking, the maximum value of parameter ξ is the length of the shortest sequence in the dataset minus one. However, when the parameter ξ is larger than 7, the model built on the features is time-consuming. To simplify the problem, we focus only on the range of ξ from 0 to 6.

Table 1 lists the results evaluated with 10-fold cross-validation by the SVM classifier on the *Saccharomyces cerevisiae* dataset. As seen in Table 1, the prediction model has the best performance when ξ = 5, achieving the higest Acc of 77.85% and the MCC of 0.5570. This indicates that when the interval between two nucleotides is equal to 5, the correlation information sufficiently reflects the inner differences between true m^6^A sites and non-m^6^A sites.

### Impact of different features

In this section, we did a further feature optimization to join 7 different individual interval features above into 329-dimension feature vector. We tested it on the *Saccharomyces cerevisiae* dataset and compared its performance with that of single interval NPPS features in the same environment. The results are listed in Table 2. As shown in Table 2, by joining all the 7 individual NPPS features, the performance is significantly improved from 77.85% to 79.92% for the Acc. This demonstrates that the correlation information of different intervals is complement to the improved predictive performance. However, simply joining features together easily generates redundant information that probably impacts the predictive performance. To validate whether there is redundant information in the joined features, we further applied three well-established feature reduction algorithms: MRMD (Maximal Relevance and Maximal Distance)^36^, RFE (Recursive Feature Elimination)^37^, and FSDI (Feature Selection based on Discernibility and Independence of a feature)^38^, to remove the redundant features from the joined NPPS features, respectively. Their results are presented in Table 2 as well. It can be seen from Table 2 that using feature reduction techniques does not improve the performance, even decreasing the performance significantly. This observation indicates the following three aspects: (1) there is very few redundant information in the joined NPPS features; (2) some important features/information are removed by using the feature reduction techniques; (3) this further confirms that the NPPS features based on different intervals contain the key correlation information that contributes together to the performance improvement.

### Comparisons with different classifiers

To verify the effectiveness of the SVM algorithm, we tested and compared the SVM algorithm with the Random Forest (RF) algorithm. The reason to choose the RF for comparison purpose is that the RF is a powerful classification algorithm, having competitive performance in several bioinformatics fields, such as DNA-binding protein prediction^39^, methylation site prediction^40^, detection of tubule boundaries^41^ and phosphorylation site prediction^42^, etc. To fairly compare the performances of SVM and RF, we performed the two algorithms under the same conditions, such as using the same joined NPPS features for modeling, and employing the same dataset for the performance evaluation. The comparison results evaluated with 10-fold cross validation are summarized in Table 3. As shown in Table 3, the SVM exhibits significantly better performance than the RF in terms of all four metrics. To be specific, the Sn, Sp, Acc, and MCC of the SVM are 79.04%, 80.80%, 79.92%, and 0.598, respectively, which are 3.37%, 4.82%, 4.10%, and 8.19% higher than that of the RF (75.67% for Sn, 75.98% for Sp, 75.82% for Acc, and 0.5165 for MCC). This indicates that the SVM algorithm is more effective than the RF algorithm for accurately identifying true m^6^A sites from non-m^6^A sites.

### Comparisons with the state-of-the-art predictors

To verify the performance of the proposed predictor, we performed and compared our predictor with state-of-the-art predictors on three benchmark datasets: the *Saccharomyces cerevisiae, Homo sapiens*, and *Arabidopsis thaliana* datasets, respectively. It should point out that the *Homo sapiens* dataset uses single interval NPPS feature for same time-consuming reason.

For the *Saccharomyces cerevisiae* dataset, we compared our predictor with the M6A-HPCS method^43^. It is worth noting that M6A-HPCS is currently the best-performing method on the *Saccharomyces cerevisiae* dataset. Thus, it is no need to compare with other methods but M6A-HPCS. Table 4 lists the jackknife results of our predictor and the M6A-HPCS method. As shown in Table 4, our predictor remarkably outperforms the M6A-HPCS method in terms of four metrics (Sn, Sp, Acc, and MCC), leading by 1.07% for Sn, 13.46% for Sp, 7.27% for Acc, and 0.14 for MCC, respectively.

For the *Arabidopsis thaliana* dataset, we compared our predictor with Chen’s method^20^. As shown in Table 5, the same rigorous jackknife test is used to assess the experiment results. We observed that our predictor obtains better performance than Chen’s method on this dataset, which further proves the effectiveness of our proposed predictor.

For the *Homo sapiens* dataset, we evaluated our predictor with the same 5-fold cross-validation test like the SRAMP predictor did^19^. We compared our predictor with the SRAMP predictor in terms of the AUROC and AUPRC. Our predictor obtained the AUROC of 0.748 and the AUPRC of 0.733, which is competitive with the SRAMP method with the AUROC of 0.797 and the AUPRC of 0.312.

In general, our predictor exhibits relatively high-level performance on three datasets cross three species. This indicates that our predictor is effective and robust for the identification of m^6^A sites cross different species.

## Conclusions

In this study, we present a novel feature encoding algorithm with multi-interval nucleotide pair position specificity, which captures not only the single RNA sequence local correlation information of multi-interval nucleotide pairs, but also the global position information, specially the global information of diversity between positive and negative samples. We test the redundant information of feature representations with the MRMD approach, optimize the SVM classifier via grid parameter searching based on F-score, and build a sequence-based predictor called RAM-NPPS for m^6^A site identification. Comparative studies on three benchmark datasets across three types of species indicate that our method is superior to the state-of-the-art methods. We establish a webserver at http://server.malab.cn/RAM-NPPS/, where users can submit uncharacterized RNA sequences and we can help to identify potential m^6^A sites within the submitted RNA sequences. In particular, the online predictor provides m^6^A site identification specific for three species: *Saccharomyces cerevisiae, Homo sapiens*, and *Arabidopsis thaliana*. It is expected that the online webserver can be a very useful tool for m^6^A site-based research. Moreover, we expect that our proposed feature representation algorithm based on multi-interval nucleotide pair position specificity can be further applied to other protein function prediction fields.

## Additional Information

**How to cite this article**: Xing, P. *et al*. Identifying N^6^-methyladenosine sites using multi-interval nucleotide pair position specificity and support vector machine. *Sci. Rep.* **7**, 46757; doi: 10.1038/srep46757 (2017).

**Publisher's note:** Springer Nature remains neutral with regard to jurisdictional claims in published maps and institutional affiliations.

## Figures and Tables

**Table 1:** Results of the proposed features by varying the parameter ξ.

ξ	Dimension	Sn (%)	Sp (%)	Acc (%)	MCC
0	50	77.35	77.66	77.51	0.5501
1	49	75.75	**79.65**	77.70	0.5544
2	48	75.29	78.50	76.89	0.5382
3	47	76.82	76.82	76.82	0.5363
4	46	75.67	77.58	76.63	0.5326
**5**	**45**	**78.12**	77.58	**77.85**	**0.5570**
6	44	76.82	77.74	77.28	0.5455

**Table 2:** Predictive results of different features.

Features	Sn (%)	Sp (%)	Acc (%)
NPPS features (ξ = 5)	78.12	77.58	77.85
Joined NPPS features	**79.04**	**80.80**	**79.92**
Joined NPPS features using MRMD	76.36	79.42	77.89
Joined NPPS features using RFE	67.18	74.50	70.84
Joined NPPS features using FSDI	67.69	75.38	71.54

**Table 3:** Performance comparison of different classifiers.

Classifiers	Sn (%)	Sp (%)	Acc (%)	MCC
Random Forest	75.67	75.98	75.82	0.5165
SVM	**79.04**	**80.80**	**79.92**	**0.5984**

**Table 4:** Comparison of identifying m^6^A sites between different methods on *Saccharomyces cerevisiae* dataset.

Predictors	Sn (%)	Sp (%)	Acc (%)	MCC	Optimized parameters
RAM-NPPS	**78.42**	**80.87**	**79.65**	**0.59**	C = 2048, γ = 0.0001220703125
M6A-HPCS	77.35	67.41	72.38	0.45	C = 8, γ = 0.0625

**Table 5:** Comparison of identifying m^6^A sites between different methods on *Arabidopsis thaliana* dataset.

Predictors	Sn (%)	Sp (%)	Acc (%)	MCC	Optimized parameters
RAM-NPPS	87.31	91.62	89.47	0.79	C = 32, γ = 0.125
Chen’s method	68.78	100.00	84.39	0.72	C = 0.5, γ = 0.0078125
